# Individual Public Transportation Accessibility is Positively Associated with Self-Reported Active Commuting

**DOI:** 10.3389/fpubh.2014.00240

**Published:** 2014-11-17

**Authors:** Sune Djurhuus, Henning Sten Hansen, Mette Aadahl, Charlotte Glümer

**Affiliations:** ^1^Department of Planning, Aalborg University, Copenhagen, Denmark; ^2^Research Centre for Prevention and Health, The Capital Region of Denmark, Glostrup, Denmark

**Keywords:** physical activity, GIS, travel planner, multi-modal network, multilevel regression

## Abstract

**Background:** Active commuters have lower risk of chronic disease. Understanding which of the, to some extent, modifiable characteristics of public transportation that facilitate its use is thus important in a public health perspective. The aim of the study was to examine the association between individual public transportation accessibility and self-reported active commuting, and whether the associations varied with commute distance, age, and gender.

**Methods:** Twenty-eight thousand nine hundred twenty-eight commuters in The Capital Region of Denmark reported self-reported time spent either walking or cycling to work or study each day and the distance to work or study. Data were obtained from the Danish National Health Survey collected in February to April 2010. Individual accessibility by public transportation was calculated using a multi-modal network in a GIS. Multilevel logistic regression was used to analyze the association between accessibility, expressed as access area, and being an active commuter.

**Results:** Public transport accessibility area based on all stops within walking and cycling distance was positively associated with being an active commuter. Distance to work, age, and gender modified the associations. Residing within 10 km commute distance and in areas of high accessibility was associated with being an active commuter and meeting the recommendations of physical activity. For the respondents above 29 years, individual public transportation accessibility was positively associated with being an active commuter. Women having high accessibility had significantly higher odds of being an active commuter compared to having a low accessibility. For men, the associations were insignificant.

**Conclusion:** This study extends the knowledge about the driving forces of using public transportation for commuting by examining the individual public transportation accessibility. Findings suggest that transportation accessibility supports active commuting and planning of improved public transit accessibility has thus a potential of providing health benefits to commuters.

## Introduction

A number of studies have found that active commuters have lower risks of a number of chronic diseases (1–4). Using public transportation involves walking or cycling to a transit stop, transfer walks, and walking to the end destination, thereby providing health benefits through regular physical activity to commuters during the week (5). Increasing the number of active commuters as an alternative to car-based commuting also has the beneficial potential of decreasing air pollution by lowering car congestion. Understanding which of the, to some extent, modifiable characteristics of public transportation that facilitate its use is thus important in a public health perspective.

Several studies have investigated the association between local access to public transportation stops and active commuting. Individual access to public transportation described by the proximity (6, 7) and density of transit stops (8–11), as well as the service frequency and number of routes at nearest stop (7) was found to be positively associated with active commuting. The access is very important because it determines how easily a person can reach the public transportation network, however, is does not quantify the accessibility by public transportation, i.e., the area and thereby opportunities than can be reached by using public transportation.

Dalvi and Martin (12) defined accessibility as the ease with which people can reach their destinations or activity sites. Thus, accessibility by public transportation describes how efficient the public transportation network is in bringing people to destinations often within a given time frame and is a widely used term in transport planning and studies of urban form. Several researchers’ have modeled individual public transportation accessibility (13–19). These models vary in complexity and some include time schedules while others rely on simplifications of the different parts of the journey, e.g., access, waiting, in-vehicle, transfer, and the egress time from the origin to destinations. Only one study has investigated individual public transportation accessibility in relation to active commuting. Frank et al. (20) found that transit accessibility was significantly associated with walk energy expenditure. Their accessibility measure described a travel survey households’ potential to reach the region’s five major activity centers. In addition, others studies on travel mode choice have found that the prevalence for car-based commuting increases with distance to work (7, 21, 22) and that there is higher prevalence for using public transportation in younger age groups (4, 23). More studies on accessibility and the association to active commuting are warranted to understand how the local public transportation is influencing active commuting.

The aim of this study was to model individual accessibility using data from a travel planner and to examine the association between individual public transportation accessibility and self-reported active commuting in The Capital Region of Denmark. Furthermore, the aim was to examine if the associations were modified by the individual commute distance, age, and gender.

## Materials and Methods

### Study population

The study included cross-sectional data collected from the Danish National Health Survey 2010 described in Christensen et al. (24). The survey contained questions on health behavior, including distance to and time spent walking or cycling to work or study each day. Respondents either completed an enclosed paper questionnaire and returning it by the mail, or online. The Capital Region of Denmark was selected as study area. The region includes Copenhagen metropolitan area as well as suburban and rural districts. From the total population above 16 years of age (1,355,000), a random sample of 95,150 was selected; the response rate was 52.3%. The data were collected from February to April 2010. The study used a subsample of 28,928 respondents living on the main island of Zealand in The Capital Region of Denmark, working or in education, between 16 and 64 years of age and with valid answers on time spent each day on active commuting in hours and minutes and individual distance to work or study. All individuals home addresses were geocoded using address matching with the official address register from the Danish Geodata Agency.

The survey was approved by the Danish Data Protection Agency. Approval from the regional Committee on Health Research Ethics was not necessary as no human biological material was included in the data collection.

### Geographical data

Public transport network data were obtained from Rejseplanen.dk, which is the official Danish travel planner search engine. The data contained information on transport mode (bus, train, s-train, metro, and ferry), routes, schedules, and geographic location of all transit stops. The schedules covered the same period as the Health Survey, i.e., February to April 2010. Road networks were obtained from the Danish Geodata agency (Kort10). Roads where walking or cycling was prohibited (e.g., motorways, highways) were excluded from the dataset before analysis.

### Multi-modal public transportation network

The geographic location of the transit stops and schedules from Rejseplanen.dk were used to construct a multi-modal transit network including all transport modes in the region (bus, trains, S-trains, metro, and ferry). In addition, road network walk links were constructed using origin-destination matrices in the Network Analyst application of ESRI ArcGIS 10.1 (Redlands, CA, USA: Environmental Systems Research Institute) from each individual home address to all public transport stops within 3 km. Walk links were also constructed between all stops not connected by a transit service to allow transfers not included in the transit network. The walk links connected stops situated no more than 1 km road network distance apart. Time spent along the access and transfer walk links were calculated from their distance and a walking speed of 5 km/h. Wait time at initial stop, in-vehicle, transfer, and egress time was integrated in the model using the time schedule. The Network Analyst application of ESRI ArcGIS 10.1 (Redlands, CA, USA: Environmental Systems Research Institute) was used to build the network having travel time as the network impedance. The interchange connections at the same stop/station at a given time and transfers were restricted by having an arrival time less than and within 20 min from the next departure time (wait time).

### Active commuting

The outcome variable was based on the self-reported time spent walking or cycling to work every day (hours, minutes) (25). The variable was dichotomized into two measures: (1) being an active commuter as binary variable (“yes” or “no”), with a cut-off value of 5 min spent on active commuting per day and; (2) meeting recommended levels of physical activity (“yes” or “no”) (≥30 min) by active commuting alone.

### Individual public transportation accessibility

**Figure 1:**
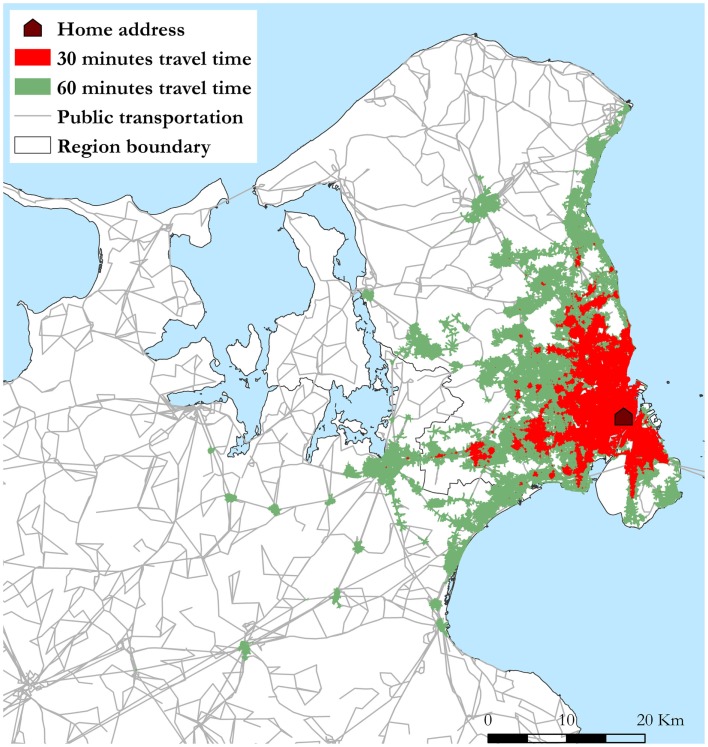
**Individual public transportation accessibility area based on entering all stops within walking (road network) distance from home address (1 km)**. The shown accessibility areas results from traveling 30 and 60 min by public transportation in all directions starting from the home address in the Copenhagen city area at 07:15 in the morning.

The individual accessibility was defined as the area each respondent can cover on the road network using active transport modes including public transportation inspired by Benenson et al. (13). The accessibility was calculated for a Monday morning between 07:15 and 08:15 during a normal week in March 2010. If no service was active within 20 min at the initial stop, the accessibility area was set equal to 0. Three measures of accessibility were created using public transportation services at (1) nearest stop within 1 km, (2) all stops within 1 km walking distance from home address, and (3) all stops within 3 km cycling distance from home address. Services at the nearest stop do not always have the best service, which is why all stops within walking distance were modeled. The 3 km access was used to capture accessibility for respondents living in the rural areas. Furthermore, the accessibility area was calculated for 30 and 60 min travel time. This was based on the assumption that 30 min travel time measures local accessibility, whereas 60 min travel time measures the regional accessibility. The 30 and 60 min travel thresholds has been used in a number of other accessibility studies (14, 26, 27). The accessible area from a given origin (*AAo*) can be expressed as *AAo* = *Aac* + *Aegr*. *Aac* is the initial access area, resulting from either 1 km walking or 3 km cycling (road network) in all directions from the individual home address. *Aegr* is the sum of egress areas, resulting from walking away from all reachable transit stops in all directions on the road network. Access and egress areas were dissolved by individual to remove overlapping areas. Individual public transportation accessibility area based on all stops within walking distance (1 km) for an individual living in Copenhagen City Center is shown in Figure 1. Ultimately, the resultant accessibility areas where divided into quartiles for each measure.

### Covariates

The individual covariates were obtained from central registers and comprised age, gender, income, and education level. Four classes of education level were defined: primary or secondary school, vocational education, academy or bachelor degree, and master’s or Ph.D. degree.

Contextual covariates (median income level, population density, and street connectivity) were aggregated by parishes, the smallest administrative units in Denmark. Street connectivity was defined by the gamma index γ = l/[3(*n* − 2)], where *n* equals the intersections (28).

### Statistical analyzes

SAS version 9.3 (SAS Institute, Inc., Cary, NC, USA) was used to perform multilevel regression analyzes (GLIMMIX procedure) to investigate if the individual public transportation accessibility was associated with being an active commuter. A two-level model was fitted with individuals (level 1, *n* = 28,928) nested within parishes (Level 2, *n* = 223).

Two empty models were estimated to detect whether there was a contextual dimension to being an active commuter and meeting recommended levels of active commuting. A three-step modeling strategy was used and ICC was calculated for each model: (1) the determinant was included in the model; (2) the individual level covariates were included to examine whether the between-parish variance was attributable to a compositional effect; (3) the parish level covariates were included to explore if the remaining between-parish variance could be explained by contextual factors. Furthermore, subgroup analyzes was conducted for distance to work expressed by living within four distance categories (≤5, >5–10, >10–20, and >20 km) from work, for age categorized in three age categories (16–29, 30–45, and 46–64 years of age) and for gender. Values of *P* < 0.05 were considered statistically significant. If an interaction was present, the odds of being an active commuter when belonging to a given distance or age category were calculated based on the full model.

## Results

Table 1 shows the descriptive statistics for the study population. 56.3% were females while 40.1% were between 46 and 64 years of age, 22.6% were between 16 and 29 and 37.3% were between 30 and 45 years of age. Approximately 73% of the study population reported daily active commuting and 50.6% reported meeting recommended levels of physical activity by active commuting (moderate physical activity). The proportion of active commuters decreased with increasing commute distance and age.

**Table 1:** **Descriptive statistics of the study population socio-demographics, home address location, and commute distance by subgroups of being an active commuter and meeting recommended levels of physical activity**.

	Total	Active commuter (≥5 min/day)		Meeting recommended levels of physical activity (≥30 min/day)	
		Yes	No	Yes	No
	*N* (%)	*N* (%)	*N* (%)	*N* (%)	*N* (%)
Total population	28,928 (100)	21,094 (72.9)	7834 (27.1)	14,629 (50.6)	14,299 (49.4)
Age (average years/SD)	40.9 (13.1)	39.7 (13.5)	44.3 (11.2)	39.3 (13.7)	42.6 (12.2)
Age groups (4 missing)
16–29 years	6538 (22.6)	5724 (87.5)	814 (12.5)	4245 (64.9)	2293 (35.1)
30–45 years	10,782 (37.3)	7507 (69.6)	3275 (30.4)	5056 (46.9)	5726 (53.1)
46–64 years	11,604 (40.1)	7860 (67.7)	3744 (32.3)	5327 (45.9)	6277 (54.1)
Gender (4 missing)
Male	12,624 (43.6)	8518 (67.5)	4106 (32.5)	5709 (45.2)	6915 (54.8)
Female	16,300 (56.3)	12,573 (77.1)	3727 (22.9)	8919 (54.7)	7381 (45.3)
Education (415 missing)
Primary or secondary school	8150 (28.2)	6434 (78.9)	1716 (21.1)	4608 (56.5)	3542 (43.5)
Vocational education	7742 (26.8)	4920 (63.5)	2822 (36.5)	3273 (42.3)	4469 (57.7)
Bachelor degree	7898 (27.3)	5822 (73.7)	2076 (26.3)	3992 (50.5)	3906 (49.5)
Master or Ph.D. degree	4723 (16.3)	3593 (76.1)	1130 (23.9)	2501 (53.0)	2222 (47.0)
Home address location
Copenhagen inner-city	9258 (32.0)	8024 (86.7)	1234 (13.3)	6396 (69.1)	2862 (31.9)
Suburban and city areas	17,477 (60.4)	11,900 (68.1)	5577 (31.9)	7607 (43.5)	9870 (56.5)
Rural	2193 (7.6)	1170 (53.4)	1023 (46.6)	626 (28.5)	1567 (71.5)
Distance to work groups
0–5 km	9237 (31.9)	7957 (86.1)	1280 (13.9)	5731 (62.0)	3506 (38.0)
>5–10 km	6676 (23.1)	5117 (76.6)	1559 (23.4)	3995 (59.8)	2681 (40.2)
>10–20 km	6516 (22.5)	4265 (65.5)	2251 (34.5)	2730 (41.9)	3786 (58.1)
>20 km	6499 (22.5)	3755 (57.8)	2744 (42.2)	2173 (33.4)	4326 (66.6)

The ICC in the two empty models showed a noticeable significant between-neighborhood variation of 13.6% in being an active commuter and 12.7% in meeting recommendations of physical activity. The ICC in the unadjusted models ranged from 3.3 to 11.6% and was significantly reduced to 0.9–1.4% in the fully adjusted models.

Table 2 shows the individual public transportation accessibility area size divided in quartiles for a travel time of 30 and 60 min. Changing the access point to the transit network from the nearest stop to all stops within 1 km increases the accessibility area in each quartile. Expanding the access to all stops within 3 km cycling, results in a maximum accessibility area of 713.3 km^2^ when the travel time is 60 min.

**Table 2:** **Quartiles of individual public transportation accessibility area for 30 and 60 min travel time calculated for all adult commuters aged 16–64 in The Capital Region of Denmark participating in the Danish National Health Survey 2010**.

	30 min travel (km^2^)	60 min travel (km^2^)
Nearest stop
Low	0–4.0	0–108.5
Medium low	4.1–19.3	108.6–313.7
Medium high	19.4–62.8	313.8–470.4
High	62.9–209.2	470.5–695.0
All stops within 1 km walking
Low	0–16.9	0–264.3
Medium low	17.0–44.5	264.4–421.2
Medium high	44.6–85.1	421.4–525.5
High	85.2–235.8	525.6–695.1
All stops within 3 km cycling
Low	0–42.0	0–383.2
Medium low	42.1–91.8	383.3–514.7
Medium high	91.9–137.9	514.8–606.4
High	138.0–235.9	606.5–713.3

The results from the multilevel regression models are shown in Table 3. No significant association was found between public transportation accessibility at nearest stop and being an active commuter. The accessibility areas, resulting from accessing all stops within walking distance were significantly positively associated with being an active commuter. An increase in accessibility area was associated with significantly higher odds of being an active commuter. The same dose–response relationship as was observed in the association between the accessibility area, resulting from accessing all stops within cycling distance and being an active commuter although there was no difference in odds of being an active commuter in the medium-low and the medium-high accessibility groups. In addition, positive associations were found between the density accessibility areas and meeting recommendations on physical activity although less pronounced compared to the associations with being an active commuter.

**Table 3:** **Crude and adjusted associations (OR) between individual public transportation accessibility area and being an active commuter and meeting recommended levels of physical activity**.

	Active commuter (≥5 min/day)		Meeting recommended levels of physical activity (≥30 min/day)	
	Model 1: crude	Model 3: fully adjusted model	Model 1: crude	Model 3: fully adjusted model
	OR (CI)	OR (CI)^a^	OR (CI)	OR (CI)^a^
Nearest stop 30 min. Acc.
Low	1.00	1.00	1.00	1.00
Medium low	**0.92 (0.85–1.00)**	0.93 (0.86–1.01)	**0.92 (0.86–1.00)**	0.93 (0.87–1.01)
Medium high	1.05 (0.96–1.14)	1.03 (0.94–1.12)	0.99 (0.91–1.06)	0.98 (0.91–1.06)
High	**1.21 (1.10–1.34)**	1.05 (0.95–1.17)	1.03 (0.95–1.12)	0.97 (0.89–1.05)
*P*-value^b^	<0.0001	0.0607	0.0832	0.3233
ICC	11.6	1.4	*11.8*	*1.3*
Nearest stop 60 min. Acc.
Low	1.00	1.00	1.00	1.00
Medium low	1.00 (0.92–1.09)	1.04 (0.96–1.13)	0.99 (0.92–1.08)	1.03 (0.95–1.12)
Medium high	1.07 (0.98–1.17)	1.03 (0.95–1.13)	1.03 (0.95–1.12)	1.04 (0.96–1.12)
High	**1.27 (1.14–1.41)**	1.07 (0.96–1.19)	1.04 (0.94–1.14)	0.97 (0.88–1.06)
*P*-value^b^	0.0002	0.6310	0.7908	0.4248
ICC	11.4	1.4	12.3	1.4
Stops within 1 km 30 min. Acc.
Low	1.00	1.00	1.00	1.00
Medium low	**1.26 (1.16–1.37)**	**1.17 (1.08–1.27)**	**1.17 (1.08–1.26)**	**1.10 (1.02–1.19)**
Medium high	**1.70 (1.54–1.87)**	**1.33 (1.21–1.47)**	**1.41 (1.29–1.55)**	**1.22 (1.11–1.33)**
High	**2.13 (1.90–2.39)**	**1.37 (1.21–1.55)**	**1.48 (1.33–1.64)**	**1.15 (1.03–1.28)**
*P*-value^b^	<0.0001	<0.0001	<0.0001	0.0005
ICC	6.2	1.2	8.4	1.1
Stops within 1 km 60 min. Acc.
Low	1.00	1.00	1.00	1.00
Medium low	**1.41 (1.28**–**1.54)**	**1.17 (1.07**–**1.28)**	**1.43 (1.31**–**1.57)**	**1.23 (1.12**–**1.34)**
Medium high	**1.90 (1.71**–**2.11)**	**1.34 (1.21**–**1.49)**	**1.82 (1.64**–**2.01)**	**1.37 (1.24**–**1.51)**
High	**2.73 (2.41**–**3.10)**	**1.44 (1.26**–**1.66)**	**2.17 (1.93**–**2.44)**	**1.36 (1.21**–**1.53)**
*P*-value^b^	<0.0001	<0.0001	<0.0001	<0.0001
ICC	4.8	1.3	5.3	0.9
Stops within 3 km 30 min. Acc.
Low	1.00	1.00	1.00	1.00
Medium low	**1.62 (1.45–1.81)**	**1.21 (1.09–1.35)**	**1.58 (1.42–1.76)**	**1.18 (1.07–1.30)**
Medium high	**2.20 (1.95–2.49)**	**1.20 (1.06–1.36)**	**2.37 (2.12–2.66)**	**1.33 (1.19–1.49)**
High	**3.36 (2.94–3.84)**	**1.44 (1.24–1.67)**	**3.03 (2.69–3.42)**	**1.42 (1.25–1.61)**
*P*-value^b^	<0.0001	<0.0001	<0.0001	<0.0001
ICC	4.2	1.3	3.6	0.9
Stops within 1 km 60 min. Acc.
Low	1.00	1.00	1.00	1.00
Medium low	**1.61 (1.44–1.80)**	**1.20 (1.07–1.33)**	**1.68 (1.51–1.87)**	**1.24 (1.12–1.37)**
Medium high	**1.98 (1.76–2.23)**	**1.19 (1.05–1.34)**	**2.11 (1.88–2.36)**	**1.28 (1.15–1.43)**
High	**3.60 (3.15–4.13)**	**1.45 (1.24–1.71)**	**3.29 (2.92–3.72)**	**1.47 (1.28–1.69)**
*P*-value^b^	<0.0001	<0.0001	<0.0001	<0.0001
ICC	3.8	1.3	3.3	0.9

*^a^Adjusted for individual age, gender, education, distance to work, and neighborhood median income, population density, and street connectivity*.

*^b^*P*-value from type III test of the association*.

*Between-neighborhood variation is expressed by intra-class correlation coefficient (ICC). Significant associations are highlighted in bold text*.

The interaction between the public transportation accessibility area and categorized commute distance was significant for all measures of accessibility (*P*-values <0.0001) as shown in Table 4.

**Table 4:** **OR table for associations between public transport accessibility and being an active commuter modified by commute distance**.

	≤5 km	>5–10 km	>10–20 km	>20 km
	OR (CI)	OR (CI)	OR (CI)	OR (CI)
Nearest stop 30 min Acc.
Low	1.00	1.00	1.00	1.00
Medium low	1.02 (0.86–1.21)	0.96 (0.81–1.14)	**0.83 (0.72–0.96)**	0.94 (0.83–1.07)
Medium high	1.08 (0.91–1.28)	**1.21 (1.03–1.43)**	0.89 (0.76–1.03)	1.00 (0.86–1.16)
High	**1.46 (1.21–1.76)**	**1.21 (1.01–1.44)**	0.87 (0.73–1.04)	**0.76 (0.63–0.91)**
*P*-value interaction = <0.0001
Nearest stop 60 min Acc.
Low	1.00	1.00	1.00	1.00
Medium low	0.98 (0.83–1.17)	1.06 (0.89–1.26)	0.93 (0.81–1.08)	**1.15 (1.00**–**1.31)**
Medium high	1.09 (0.91–1.29)	1.14 (0.96–1.34)	0.92 (0.79–1.07)	1.01 (0.87–1.18)
High	**1.46 (1.21**–**1.77)**	**1.24 (1.04**–**1.49)**	0.85 (0.70–1.02)	**0.75 (0.62**–**0.91)**
*P*-value interaction = <0.0001
Stops within walking distance 30 min Acc. (1 km)
Low	1.00	1.00	1.00	1.00
Medium low	**1.26 (1.05**–**1.50)**	**1.23 (1.03**–**1.46)**	**1.20 (1.05**–**1.39)**	1.08 (0.95–1.23)
Medium high	**1.56 (1.30**–**1.87)**	**1.35 (1.13**–**1.61)**	**1.33 (1.14**–**1.56)**	**1.25 (1.06**–**1.47)**
High	**1.94 (1.58**–**2.37)**	**1.63 (1.34**–**1.99)**	1.19 (0.99–1.44)	0.87 (0.71–1.06)
*P*-value interaction = <0.0001
Stops within walking distance 60 min Acc. (1 km)
Low	1.00	1.00	1.00	1.00
Medium low	**1.31 (1.09**–**1.56)**	**1.25 (1.04**–**1.50)**	**1.24 (1.07**–**1.43)**	1.05 (0.92–1.21)
Medium high	**1.54 (1.28**–**1.86)**	**1.49 (1.24**–**1.80)**	**1.37 (1.17**–**1.61)**	1.18 (1.00–1.40)
High	**2.24 (1.80**–**2.78)**	**1.72 (1.40**–**2.12)**	**1.29 (1.06**–**1.58)**	0.89 (0.72–1.09)
*P*-value interaction = <0.0001
Stops within walking distance 30 min Acc. (3 km)
Low	1.00	1.00	1.00	1.00
Medium low	1.12 (0.93–1.36)	**1.22 (1.00–1.48)**	**1.40 (1.20–1.64)**	**1.18 (1.02–1.36)**
Medium high	**1.34 (1.10–1.64)**	**1.39 (1.15–1.69)**	**1.31 (1.10–1.56)**	0.85 (0.70–1.02)
High	**2.01 (1.60–2.51)**	**1.79 (1.43–2.24)**	**1.31 (1.06–1.62)**	0.85 (0.69–1.06)
*P*-value interaction = <0.0001
Stops within walking distance 60 min Acc. (3 km)
Low	1.00	1.00	1.00	1.00
Medium low	1.15 (0.95–1.39)	**1.24 (1.02–1.51)**	**1.42 (1.21–1.67)**	1.12 (0.96–1.29)
Medium high	**1.28 (1.05–1.55)**	**1.40 (1.15–1.69)**	**1.34 (1.13–1.59)**	0.93 (0.78–1.11)
High	**2.14 (1.70–2.71)**	**1.97 (1.56–2.48)**	**1.25 (1.00–1.56)**	**0.79 (0.63–0.98)**
*P*-value interaction = <0.0001				

*Significant associations are highlighted in bold text*.

For the accessibility areas, resulting from 1 km walking or 3 km cycling, an increase in accessibility area was associated with significantly higher odds of being an active commuter. For commuters having between 10 and 20 km commute distance, an increase in the accessibility area (1 km walking and 3 km cycling) was associated with significantly higher odds of being an active commuter in the medium-low quartile of accessibility compared to low accessibility. Living more than 20 km from work, the association between public transportation accessibility and being an active commuter became insignificant and even negative for medium high and high accessibility in the model with all stops within 3 km cycling. Positive significant associations were also found between all density measures and meeting recommendations of physical activity for participants with commute distance of ≤10 km. The associations were strongest for those having between 5 and 10 km commute distance. For participants having between 10 and 20 km commute distance, a medium-low or medium-high accessibility based on 1 km walking or 3 km cycling was associated with significantly higher odds of meeting recommendations of physical activity compared to having low-public transportation accessibility. For those having more than 20 km commute distance, accessibility area was not associated with meeting recommended levels of physical activity.

The subgroup analysis with age showed that for the age category 16–29 years, the association between accessibility (1 km walking and 3 km cycling) and being an active commuter was insignificant (results not shown). For the respondents in the other two age groups, 30–45 and 46–64 years, the accessibility was positively associated with being an active commuter. The association was strongest among the 30–45 years old. The subgroup analysis with age showed the same results with meeting recommended levels of physical activity.

For women, there was a significant positive association between accessibility area based on all stops within walking and cycling distance and being an active commuter (results not shown). Furthermore, women having high accessibility based on services at the nearest stop (30 and 60 min) had significantly higher odds of being an active commuter compared to the reference group (low). For men, the associations were insignificant. For women, there was a significantly positive association between accessibility area based on all stops within walking and cycling distance and meeting recommendations of physical activity. No significant associations were found for women between accessibility based on services at the nearest stop (30 and 60 min) and meeting recommendations of physical activity. For men, the associations were less pronounced, although suggesting that higher accessibility based on walking and cycling was positively associated with meeting recommendations of physical activity.

## Discussion

The findings suggest that individual public transportation accessibility is associated with commuters travel preferences and higher public transportation accessibility is associated with being an active commuter and meeting recommended levels of physical activity from active commuting only. The study adds to the previous studies of the access to public transportation and associated active commuting by combining the access to public transportation, i.e., density of stops, service frequency, and available routes with the efficiency of the public transportation network in enabling the respondent in reaching destinations. The study further highlights the difference in accessibility and the association with active commuting between using services only at the nearest stop and at all stops within walking or biking distance.

Those living in the metropolitan and inner suburban areas often have multiple transit stops within walking distance that provide different transit services and modes. The insignificant association found between individual public transportation accessibility and active commuting for public transportation accessibility at the nearest stop may thus be explained by the fact that the nearest stop provides a too simplified picture of the “real” public transportation accessibility. Another explanation may be due to the way the accessibility is modeled. The nearest stop measure is quite sensitive to services leaving between the time a participant enters the stop until the last allowed departure time at 07:35. This can result in accessibility areas of 0 km^2^ although services may leave at 07:36 and thereby lower the variance of the measure. Commuters tend to optimize their trip by entering a station just in time for the service to depart. This cannot be captured in this analysis.

The positive association found between accessibility based on all stops within walking distance (1 km) and active commuting reflect other findings that accessible and efficient public transportation is conducive for being an active commuter (7–11, 20).

In accordance with other studies, we found that distance to work or study influence active commuting (7, 21, 22). Living close to work (within 10 km) in areas of high-public transportation accessibility are associated with being an active commuter. Metropolitan and city areas have high-public transportation accessibility, high density of opportunities such as jobs and a supportive infrastructure that promotes walking or cycling and use of public transportation. There is prevalence for car-based commuting at commute distances longer than 20 km even if public transportation accessibility is high, resulting in a negative association between public transportation accessibility and active commuting.

A high proportion of the respondents between 16 and 29 live close to their work or study and walk or cycle all the way. This weakens the effect of public transportation (the association is insignificant) although other studies find that this age group is the most inclined to use public transportation to travel (4, 23). For the other age groups, the positive associations found reflect that using active commute modes becomes more attractive if the potential for reaching other destinations is high.

The results suggest that men’s active commute patterns are less influenced by public transportation than women, which may be caused by more car-based commuting. Living in areas of high accessibility is not associated with active commuting in men whereas women show a clear dose–response relationship between accessibility and the odds for being an active commuter.

Higher public transportation accessibility has the potential for increasing active commuting and thereby providing important health benefits through active transportation. Future transport planning should evaluate how longer commute trips (>10 km) can be covered by better public transportation services to create an alternative to car-based commuting. Save roads and bike lanes to and from stations/stops, allowing bikes to be transported on trains and in busses and more flexible transfers between stops not connected by services are all things that make public transportation services more available by reducing access and egress time. Public transportation service level could be improved with shorter wait time between services and optimal transfer time. This will increase accessibility by public transportation. Furthermore, it should include restrictions on car-based commuting such as restrictions on car-park facilities, which have a positive impact toward active commuting in Denmark (22).

Accessibility has only been investigated in association to active commuting by Frank et al. (20). This is to our knowledge the first study looking at the association between accessibility area and active commuting. Accessibility is directly linked to the local public transportation network. Whether accessibility in other countries shows the same association with active commuting is yet to be explored. In this study, accessibility is calculated using a simple two-dimensional approach and time tables. Higher availability of more disaggregate data and new approaches to calculating accessibility using web-crawlers or journey planner API’s (19) will undoubtedly increase research within accessibility and active commuting.

### Strengths and limitations

This study has a number of strengths. The multi-modal network constructed with integrated time schedule made it possible to calculate individual public transportation accessibility based on network travel time and walking along the road network. The accessibility measure includes the potential to travel in the association analysis in contrast to just looking at the access to public transportation stops. The large study population selected from one of the largest health surveys in the world and the individual register-based socioeconomic data provide a unique study base. The multilevel model accounted for the large neighborhood effect found.

There are a number of limitations to this study. The cross-sectional design makes it impossible to draw conclusion on causality. The self-reported daily active commuting may be subject to information bias. The active commuting information is restricted to time spent walking or cycling to work or study, and it does not refer to time spent in usage of public transportation or car. The high proportion of respondents reporting active commuting in this study is substantially higher than in other studies. It is therefore unknown whether the results may be generalizable to other countries or cities where active commuting is not as common. The multi-modal network uses the time schedule to calculate travel time but no information about service performance have been included. No land-use parameter such as reachable jobs is included in the individual public transportation accessibility meaning that all areas are weighted equally important when commuting. The public transportation accessibility is thus used as a measure of how efficient the public transportation system is in bringing respondents to other destinations. Transfers between transport modes were not limited in this study although this is often listed as an inconvenience when using public transportation (29). Further work would benefit from including work addresses in order to model routes to work using different transport modes and examine associated travel choices.

## Conclusion

This study extends the knowledge about the driving forces of using public transportation for commuting by examining the individual public transportation accessibility. The findings suggest that provision of good public transportation accessibility is associated with active commuting although it varies with distance to work or study, age, and gender. The implication for future transport and health policy is to improve public transit services by increasing accessibility through improved access and linkage between services and keep travel costs at a rational level.

## Author Contributions

All authors designed the protocol for this study. Sune Djurhuus performed the GIS and statistical analysis and drafted the manuscript. Henning Sten Hansen, Mette Aadahl, and Charlotte Glümer critically revised and helped to draft the manuscript. All authors read and approved the final manuscript.

## Conflict of Interest Statement

The authors declare that the research was conducted in the absence of any commercial or financial relationships that could be construed as a potential conflict of interest.

## References

[1] Bauman AE Reis RS Sallis JF Wells JC Loos RJ Martin BW. Correlates of physical activity: why are some people physically active and others not? Lancet (2012) 380(9838):258–71. 10.1016/S0140-6736(12)60735-1 22818938

[2] Gordon-Larsen P Boone-Heinonen J Sidney S Sternfeld B Jacobs DR Jr Lewis CE. Active commuting and cardiovascular disease risk: the cardia study. Arch Intern Med (2009) 169(13):1216–23. 10.1001/archinternmed.2009.163 19597071 PMC2736383

[3] Hamer M Chida Y. Active commuting and cardiovascular risk: a meta-analytic review. Prev Med (2008) 46(1):9–13. 10.1016/j.ypmed.2007.03.006 17475317

[4] Laverty AA Mindell JS Webb EA Millett C. Active travel to work and cardiovascular risk factors in the United Kingdom. Am J Prev Med (2013) 45(3):282–8. 10.1016/j.amepre.2013.04.012 23953354

[5] Pratt M Sarmiento OL Montes F Ogilvie D Marcus BH Perez LG The implications of megatrends in information and communication technology and transportation for changes in global physical activity. Lancet (2012) 380(9838):282–93. 10.1016/S0140-6736(12)60736-3 22818940 PMC4843126

[6] Coogan PF White LF Adler TJ Hathaway KM Palmer JR Rosenberg L. Prospective study of urban form and physical activity in the Black Women’s Health Study. Am J Epidemiol (2009) 170(9):1105–17. 10.1093/aje/kwp264 19808635 PMC3031349

[7] Dalton AM Jones AP Panter JR Ogilvie D. Neighbourhood, route and workplace-related environmental characteristics predict adults’ mode of travel to work. PLoS One (2013) 8(6):e67575. 10.1371/journal.pone.0067575 23840743 PMC3686740

[8] Hino AA Reis RS Sarmiento OL Parra DC Brownson RC. Built environment and physical activity for transportation in adults from Curitiba, Brazil. J Urban Health (2013) 91(3):446–62. 10.1007/s11524-013-9831-x 24096625 PMC4074327

[9] Li F Harmer PA Cardinal BJ Bosworth M Acock A Johnson-Shelton D Built environment, adiposity, and physical activity in adults aged 50-75. Am J Prev Med (2008) 35(1):38–46. 10.1016/j.amepre.2008.03.021 18541175 PMC2459142

[10] Lovasi GS Neckerman KM Quinn JW Weiss CC Rundle A Effect of individual or neighborhood disadvantage on the association between neighborhood walkability and body mass index. Am J Public Health (2009) 99(2):279–84 10.2105/AJPH.2008.138230 19059849 PMC2622783

[11] McConville ME Rodriguez DA Clifton K Cho G Fleischhacker S. Disaggregate land uses and walking. Am J Prev Med (2011) 40(1):25–32. 10.1016/j.amepre.2010.09.023 21146764

[12] Dalvi MQ Martin KM The measurements of accessibility: some preliminary results. Transportation (1978) 5:17–42 10.1007/BF00165245

[13] Benenson I Martens K Rofé Y Kwartler A Public transport versus private car GIS-based estimation of accessibility applied to the Tel Aviv metropolitan area. Ann Reg Sci (2011) 47(3):499–515 10.1007/s00168-010-0392-6

[14] Gent C Symonds G Advances in public transportation accessibility assessments for development control – a proposed methodology. PTRC Annual Transport Practitioners’ Meeting. London, UK (2005).

[15] Lei TL Church RL Mapping transit-based access: integrating GIS, routes and schedules. Int J Geogr Inf Sci (2010) 24(2):283–304 10.1080/13658810902835404

[16] Liu S Zhu X Accessibility analyst: an integrated GIS tool for accessibility analysis in urban transportation planning. Environ Plann B Plann Des (2004) 31(1):105–24 10.1068/b305

[17] Mavoa S Witen K McCreanor T O’Sullivan D GIS based destination accessibility via public transit and walking in Auckland, New Zealand. J Transp Geogr (2012) 20:15–22 10.1016/j.trangeo.2011.10.001

[18] O’Sullivan D Morrison A Shearer J Using desktop GIS for the investigation of accessibility by public transport: an isochrone approach. Int J Geogra Inf Sci (2000) 14(1):85–104 10.1080/136588100240976

[19] Salonen M Toivonen T Modelling travel time in urban networks: comparable measures for private car and public transport. J Transp Geogr (2013) 31:143–53 10.1016/j.jtrangeo.2013.06.011

[20] Frank LD Greenwald MJ Winkelman S Chapman J Kavage S. Carbonless footprints: promoting health and climate stabilization through active transportation. Prev Med (2010) 50(Suppl 1):S99–105. 10.1016/j.ypmed.2009.09.025 19850071

[21] Badland HM Schofield GM Garrett N. Travel behavior and objectively measured urban design variables: associations for adults traveling to work. Health Place (2008) 14(1):85–95. 10.1016/j.healthplace.2007.05.002 17590378

[22] DTU. The Danish National Travel Survey (2013). Available from: http://www.modelcenter.transport.www6.sitecore.dtu.dk/english/TU/Hovedresultater

[23] Morency C Trépanier M Demers M Walking to transit: an unexpected source of physical activity. Transport Pol (2011) 18:800–6 10.1016/j.tranpol.2011.03.010

[24] Christensen AI Ekholm O Glumer C Andreasen AH Hvidberg MF Kristensen PL The Danish National Health Survey 2010. study design and respondent characteristics. Scand J Public Health (2012) 40(4):391–7. 10.1177/1403494812451412 22786925

[25] Andersen LG Groenvold M Jorgensen T Aadahl M. Construct validity of a revised physical activity scale and testing by cognitive interviewing. Scand J Public Health (2010) 38(7):707–14. 10.1177/1403494810380099 20823047

[26] Kawabata M Spatiotemporal dimensions of modal accessibility disparity in Boston and San Francisco. Environ Plan A (2009) 41:183–98 10.1068/a4068

[27] Kawabata M Shen Q Job accessibility as an indicator of auto-oriented urban structure: a comparison of Boston and Los Angeles with Tokyo. Environ Plann B Plann Des (2014) 33:115–30 10.1068/b31144

[28] Berrigan D Pickle LW Dill J. Associations between street connectivity and active transportation. Int J Health Geogr (2010) 9:20. 10.1186/1476-072X-9-20 20412597 PMC2876088

[29] Stone J Mees P Planning public transport networks in the post-petroleum era. Aust Plan (2010) 47:263–71 10.1080/07293682.2010.526550

